# Diagnostic performance of Kaiser score, ADC, and their combination for BI-RADS 4 breast lesions: subgroup and misclassification analysis

**DOI:** 10.3389/fonc.2026.1802360

**Published:** 2026-07-23

**Authors:** Hui Zhou, Ling Wei, Zhiqin Zhou, Guoyuan Jiang, Shiguang Li, Xianchun Zeng

**Affiliations:** 1 Department of Radiology, The Affiliated Jinyang Hospital of Guizhou Medical University (The Second People's Hospital of Guiyang), Guiyang, China; 2 The First People’s Hospital of Zunyi, Zunyi, China; 3 Department of Medical Imaging, The First People's Hospital of Zunyi, The Third Affiliated Hospital of Zunyi Medical University, Zunyi, China; 4 Breast and Thyroid Surgery Center, The First People's Hospital of Zunyi, The Third Affiliated Hospital of Zunyi Medical University, Zunyi, China; 5 Department of Nuclear Medicine, Guizhou Provincial People's Hospital, Guiyang, China

**Keywords:** breast MRI, BI-RADS 4, Kaiser score, apparent diffusion coefficient (ADC), misclassification analysis, diagnostic accuracy

## Abstract

**Background:**

The Kaiser score and apparent diffusion coefficient (ADC) have been proposed as diagnostic adjuncts to reduce unnecessary biopsies in Breast Imaging Reporting and Data System (BI-RADS) 4 breast lesions. However, which specific histopathologic entities are prone to misclassification by each diagnostic model remains poorly characterized, limiting clinical guidance for borderline cases.

**Objective:**

To compare the diagnostic performance of the Kaiser score, ADC, and their combination (Kaiser+) stratified by lesion morphology, and to identify histopathologic subtypes most susceptible to misclassification.

**Methods:**

This retrospective study included 222 patients with BI-RADS 4 breast lesions confirmed by histopathology. Two radiologists independently evaluated lesions using the Kaiser score and ADC values (threshold: 1.4 × 10^-3^ mm²/s). Diagnostic performance was assessed using receiver operating characteristic (ROC) analysis, and misclassification patterns were analyzed by pathologic subtype using McNemar’s test with Bonferroni correction.

**Results:**

The Kaiser score achieved the highest area under the curve (AUC) of 0.901 (95% CI: 0.856–0.941), followed by Kaiser+ (AUC 0.890; 95% CI: 0.845–0.930) and ADC alone (AUC 0.733; 95% CI: 0.666–0.800; *p* < 0.001 vs. Kaiser). Kaiser demonstrated superior performance in mass lesions (AUC 0.926; 95% CI: 0.878–0.965) compared with non-mass enhancement (NME) (AUC 0.801; 95% CI: 0.681–0.905). Pathology-stratified analysis revealed distinct misclassification patterns: fibroadenoma, breast hyperplasia, and intraductal papilloma accounted for most FP cases, with ADC showing significantly higher FP rates (66.1%; 95% CI: 57.3%–73.9%) than Kaiser (19.8%; 95% CI: 13.7%–27.8%) or Kaiser+ (17.4%; 95% CI: 11.6%–25.1%; *p*<0.001). Among malignant lesions, ductal carcinoma *in situ* (DCIS) and mucinous carcinoma were the majority of frequently misclassified as FN across all models.

**Conclusion:**

The Kaiser score outperformed ADC alone in BI-RADS 4 lesions. Pathology-stratified analysis identified subtype-specific diagnostic pitfalls: benign cellular and proliferative lesions drive FP cases (especially with ADC), while low-conspicuity malignancies (DCIS, mucinous carcinoma) drive FN cases. Awareness of these patterns may guide individualized biopsy decisions in clinical practice.

## 1 Introduction

The Breast Imaging Reporting and Data System (BI-RADS) is a standardized lexicon developed by the American College of Radiology to unify terminology and risk stratification across breast imaging modalities (1). Among its categories, BI-RADS category 4 lesions represent a diagnostic gray zone, with malignancy probabilities ranging from 2% to 95% and considerable inter-reader variability in assessment (2, 3). These factors frequently lead to biopsy recommendations, even though a substantial proportion of lesions ultimately prove benign. Consequently, patients are frequently subjected to unnecessary invasive procedures, psychological stress, and increased healthcare burden (4).

Diffusion-weighted imaging (DWI) and its quantitative parameter, the apparent diffusion coefficient (ADC), have emerged as valuable adjuncts in breast magnetic resonance imaging (MRI) for differentiating benign from malignant lesions. Prior studies have demonstrated that ADC thresholds approximating 1.5 × 10^-3^ mm²/s could reduce unnecessary biopsies without compromising sensitivity (5). Nevertheless, diagnostic challenges persist for specific histologic subtypes, such as fibroadenomas and mucinous carcinomas, where overlapping diffusion characteristics may limit discriminatory power (6, 7).

To improve diagnostic consistency and reduce unnecessary biopsies, the Kaiser score was developed as a structured decision-making algorithm based on five dynamic contrast-enhanced MRI (DCE-MRI) features: lesion margin, internal enhancement pattern, time–intensity curve type, presence of perilesional edema, and presence of a root sign (8). This structured algorithm guides malignancy risk stratification, particularly for lesions categorized as BI-RADS 4. several studies have reported promising diagnostic performance when ADC is integrated with the Kaiser score (9–12).

While some studies have evaluated performance across lesion morphologies (12), the benefit of the Kaiser score appears to be morphology-dependent and may be attenuated in specific histologic subtypes (13). However, a dedicated subtype-stratified analysis identifying which specific pathologic entities are prone to misclassification with each diagnostic model remains lacking, limiting our understanding of when and why these tools may fail in clinical practice (14).

Building on prior work demonstrating overall diagnostic improvements with ADC integration (8, 9), the specific histopathologic entities most susceptible to misclassification remain inadequately characterized. To address this knowledge gap, we validated a Kaiser score–ADC combined classification workflow in an independent cohort of BI-RADS 4 lesions and conducted a comprehensive pathology–stratified analysis of diagnostic errors. Our study had three primary objectives: (i) to compare diagnostic performance stratified by lesion morphology (mass versus non-mass enhancement); (ii) to identify which benign and malignant histopathologic subtypes are most frequently misclassified by each diagnostic model; and (iii) to determine whether ADC integration modifies the sensitivity-specificity trade-off across different lesion subtypes.

## 2 Materials and methods

### 2.1 Study population

**Figure 1:**
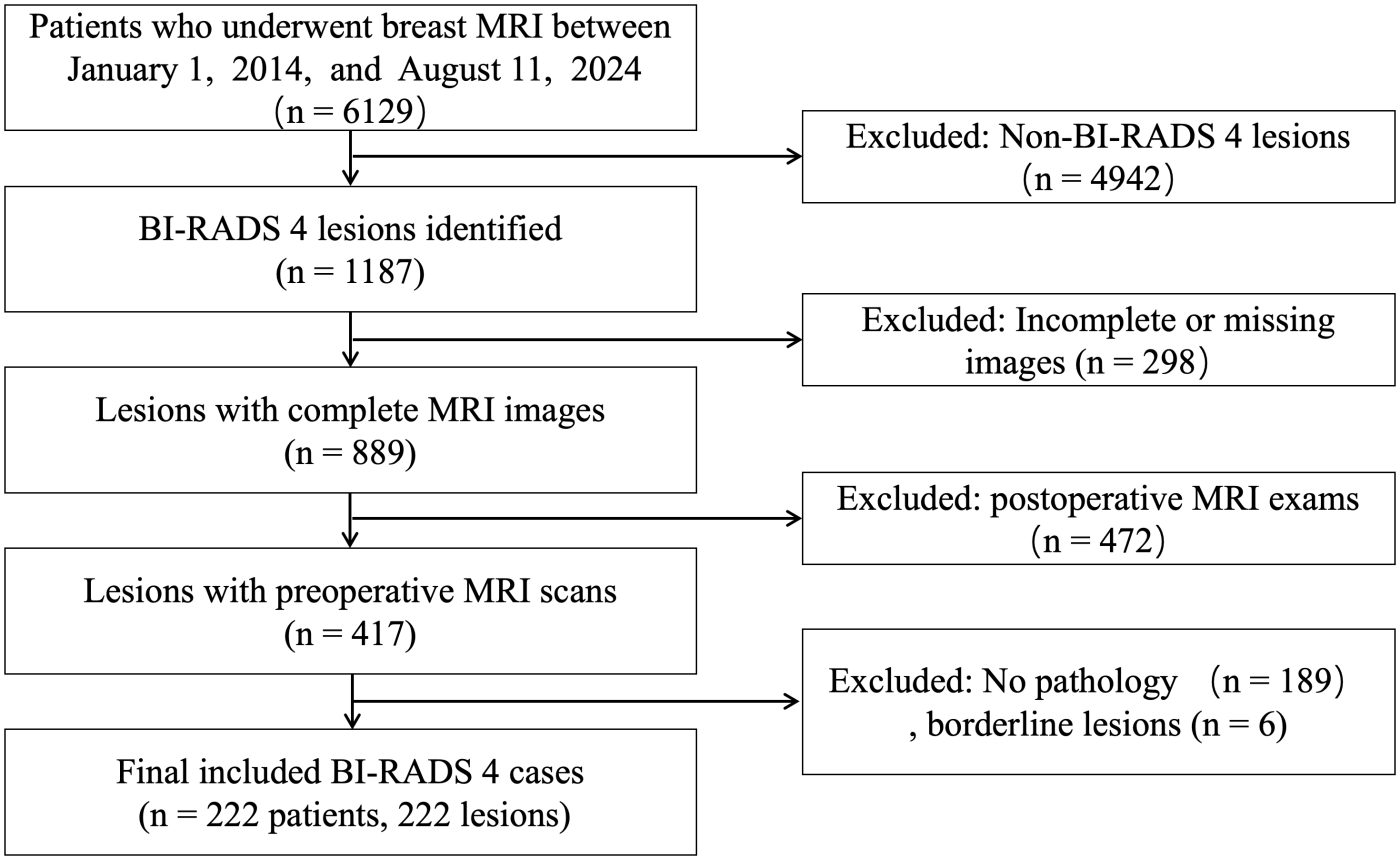
Flowchart of patient selection. 6129 patients who underwent breast MRI were reviewed between January 1, 2014, and August 11, 2024. After applying inclusion and exclusion criteria, 222 BI-RADS 4 lesions in 222 patients were included for final analysis.

Female patients who underwent breast MRI examinations and received BI-RADS category 4 classifications at a tertiary hospital between January 2014 and August 2024 were retrospectively reviewed. The inclusion criteria were (1): MRI report indicating a BI-RADS category 4 lesion; (2) availability of complete MRI Digital Imaging and Communications in Medicine (DICOM) data sufficient for Kaiser score evaluation and ADC measurement; (3) histopathological confirmation of diagnosis. In cases of multiple BI-RADS 4 lesions, only one lesion per patient was included to avoid clustering effects. The exclusion criteria were: (1) missing or poor-quality MRI images; (2) prior breast surgery with postoperative MRI findings; (3) pathological diagnosis of borderline tumors; (4) lesions identified after initiation of chemotherapy; and (5) presence of other concurrent malignancies. A total of 6,129 patients initially screened, 222 patients with 222 BI-RADS 4 lesions (one lesion per patient) were ultimately included. The detailed patient selection process is illustrated in **Figure 1**.

### 2.2 MRI protocol

All MRI examinations were performed using either a 1.5-T Avanto or a 3.0-T Skyra scanner (Siemens Healthcare, Erlangen, Germany), both were equipped with dedicated bilateral breast coils. Given the retrospective nature of this 10-year study (2014 - 2024), the precise distribution of examinations across field strengths could not be determined; however, standardized acquisition protocols were maintained throughout the study period (**Supplementary Table 1**).

The imaging protocol included axial T1-weighted imaging (T1WI), T2-weighted imaging (T2WI), DWI, and DCE-MRI. DWI was acquired using b-values of 0 and 800 s/mm², and ADC maps were automatically generated by the scanner software. DCE-MRI was performed following intravenous administration of a gadolinium-based contrast agent. Detailed acquisition parameters are provided in **Supplementary Table 1**. An ADC threshold of 1.4 × 10^-3^ mm²/s was applied uniformly across both field strengths, consistent with validated multicenter protocols (15).

### 2.3 MRI image assessment and diagnostic model definitions

**Figure 2:**
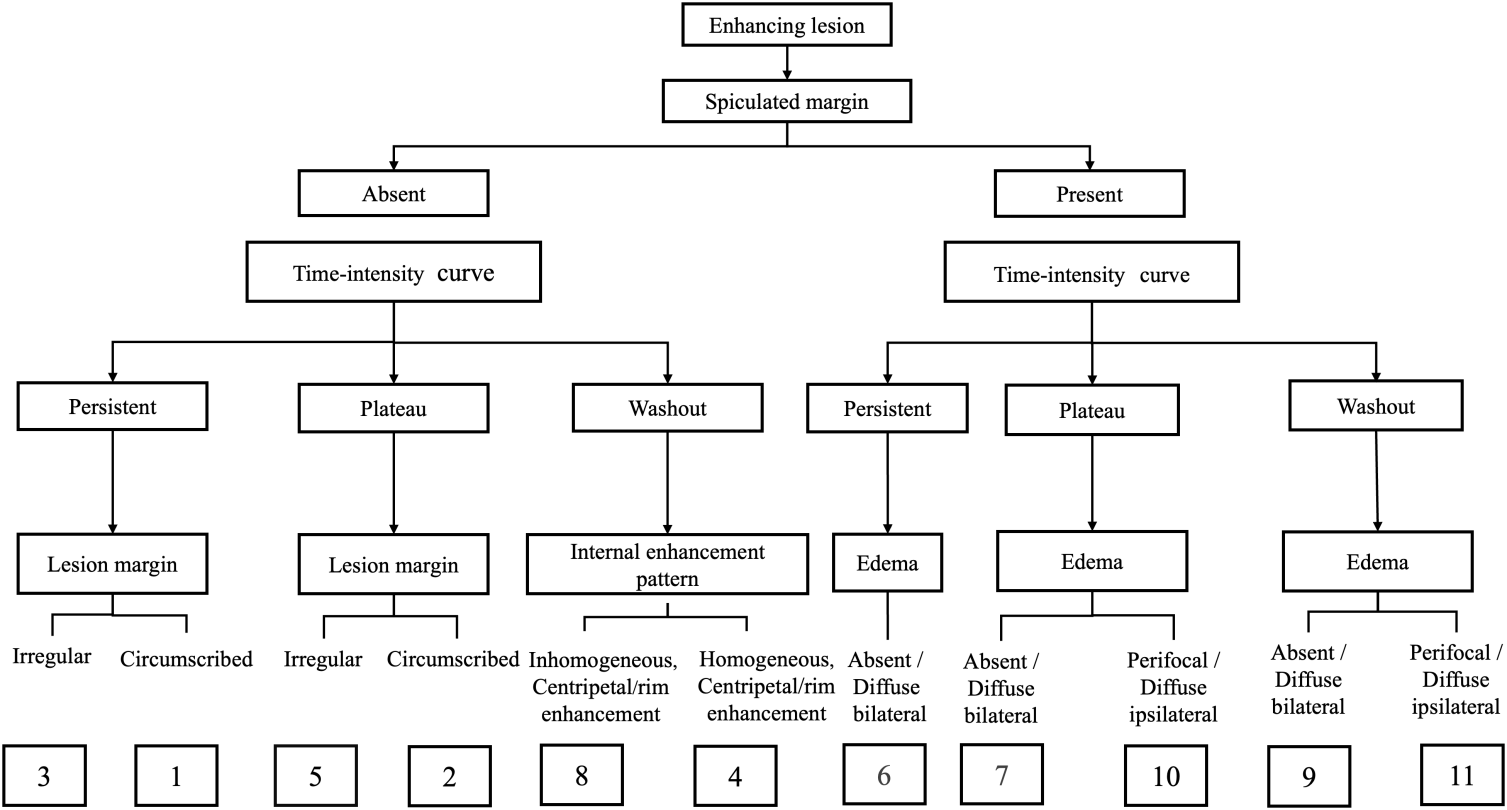
Kaiser scoring algorithm flowchart. This flowchart illustrates the Kaiser scoring process based on five multiparametric MRI features. The scoring integrates assessment of spiculated margin, time–intensity curve pattern, lesion margin or internal enhancement pattern, and presence and distribution of peritumoral edema. Each diagnostic pathway leads to a final numerical score ranging from 1 to 11, corresponding to the likelihood of malignancy. In clinical practice, scores ≥5 typically indicate malignant potential, while scores ≥7 are associated with a high probability of malignancy and often guide biopsy recommendations.

All MRI images were independently reviewed by two dedicated breast radiologists (Reader 1 with 12 years of experience, and Reader 2 with 8 years of experience in breast MRI), who blinded to the histopathological results. Discrepancies in Kaiser scores (differing by ≥1 point) or ADC measurements (differing by >10%) were resolved through in-person consensus discussion between the two readers; when agreement could not be reached, a third senior radiologist (with more than20 years of experience in breast MRI) served as the final arbiter. Final Kaiser scores (**Figure 2**) and ADC measurements used for all diagnostic performance analyses were derived from consensus readings.

Three diagnostic models were evaluated in this study:

Kaiser score–based model: The Kaiser score is a structured diagnostic algorithm based on five DCE-MRI features: lesion margin, internal enhancement pattern, time–intensity curve type, presence of perilesional edema, and presence of a root sign (8). Per the original Kaiser score publication (16), scores below 5 are considered benign, while a score of ≥5 indicates malignant potential and biopsy is recommended.

Apparent diffusion coefficient–based model: ADC values were measured from automatically generated ADC maps using a circular region of interest (ROI) manually placed within the most solid and enhancing portion of the lesion on the largest axial slice. Although individual reader ADC measurements were not retained separately, ADC is an objective quantitative metric with well-established inter-reader reproducibility in breast MRI, as demonstrated by the multicenter American College of Radiology Imaging Network (ACRIN) 6698 trial (interreader intraclass correlation coefficient (ICC) = 0.92; 95% confidence interval (CI): 0.80–0.97) (17). Areas of necrosis, hemorrhage, cystic degeneration, non-enhancing tissue, surrounding normal parenchyma, and adjacent large vessels were carefully avoided to minimize partial volume effects. An ADC threshold of 1.4 × 10^-3^ mm²/s was adopted from the model proposed by Baltzer et al. (18) and validated in multicenter studies (15).

Kaiser score adjusted by ADC (Kaiser+): The Kaiser+ model integrates ADC values with the Kaiser score as previously described (8, 18). When ADC ≥1.4 × 10^-3^ mm²/s, 4 points are subtracted from the Kaiser score based on the rationale that lesions exhibiting high ADC values demonstrate diffusion characteristics more consistent with benign pathology (16). This ADC-based adjustment has been validated across multiple studies and is recommended as an optional adjunct to the Kaiser score to improve specificity without compromising sensitivity. It should be noted that this Kaiser+ formulation represents a purely MRI-based integration and does not incorporate mammographic features such as microcalcifications, which are described as an additional optional moderator in the original publication (16) but are primarily assessed on mammography rather than MRI.

### 2.4 Reference standard

Histopathological evaluation served as the reference standard for all diagnostic assessments. All lesions were histopathologically confirmed through either image-guided core needle biopsy or surgical excision. All pathology slides were independently reviewed by two board-certified breast pathologists. In cases of discordant diagnoses, the disagreement was first addressed through direct discussion between the two pathologists; when consensus could not be reached, a third senior pathologist with more than 20 years of experience served as the final arbiter. For lesions classified as high-risk, including atypical ductal hyperplasia and papillary lesions with atypia, definitive diagnoses were established through surgical excision.

### 2.5 Statistical analysis

All statistical analyses were performed using SPSS (version 26.0; IBM Corp., Armonk, NY, USA) and R (version 4.5.0; R Foundation for Statistical Computing, Vienna, Austria). Inter-reader agreement for Kaiser scores was assessed using the ICC (two-way mixed-effects model, absolute agreement type) calculated using the irr package in R, with ICC values interpreted as: poor (<0.50), moderate (0.50–0.75), good (0.75–0.90), and excellent (>0.90) (19). Continuous variables were compared using the t-test or Mann–Whitney U test; categorical variables were compared using chi-square or Fisher’s exact test. Diagnostic performance was assessed through ROC curve analysis (AUC with 95% CI). The Youden index was used to determine the optimal operating point and the optimal Kaiser score threshold. Three-way AUC comparisons were performed using DeLong tests with Bonferroni correction. Decision curve analysis (DCA) was performed to evaluate the clinical utility of each diagnostic model. Positive likelihood ratio (PLR) and negative likelihood ratio (NLR) were calculated as prevalence-independent measures of diagnostic accuracy, using the log method with 95% CI.

Subtype-stratified diagnostic error analysis: Using histopathology as the reference standard, false positives (FP) were defined as benign lesions classified as malignant, and false negatives (FN) were defined as malignant lesions classified as benign under each model’s prespecified thresholds (Kaiser/Kaiser+ ≥5; ADC ≤1.4×10^-3^ mm²/s). Pairwise comparisons of FP and FN rates were performed using McNemar’s test with Bonferroni correction (adjusted α = 0.017 for three pairwise comparisons), with raw and Bonferroni-adjusted p-values reported. For all other comparisons, a two-sided α of 0.05 was considered statistically significant.

## 3 Results

### 3.1 Baseline clinical and pathological characteristics

A total of 222 patients with 222 BI-RADS 4 breast lesions (one lesion per patient) were included in the final analysis. The mean age was 47.6 ± 11.1 years (range, 16–76 years). The cohort comprised 219 female (98.6%) and 3 male (1.35%) patients. Of the 222 lesions, 128 (57.7%) were located in the left breast and 94 (42.3%) in the right breast. Regarding lesion morphology, 165 (74.3%) were mass lesions and 57 (25.7%) were non-mass enhancement (NME) lesions. Histopathology revealed 121 (54.5%) benign and 101 (45.5%) malignant lesions. Among benign lesions, fibroadenoma was the most common (n = 54, 44.6%), followed by breast hyperplasia (n = 39, 32.2%) and intraductal papilloma (n = 20, 16.5%); other benign lesions accounted for the remaining 8 cases (6.6%). Among malignant lesions, invasive ductal carcinoma (IDC) predominated (n=58, 57.4%) followed by ductal carcinoma *in situ* (DCIS) (n=21, 20.8%) and mucinous carcinoma (n=6, 5.9%); other malignant lesions accounted for the remaining 16 cases (15.8%). Patients with malignant lesions were significantly older than those with benign lesions (51.4 ± 11.6 vs. 44.5 ± 9.7 years; *p* < 0.001). No significant between-group differences were observed for sex (*p* = 0.592, Fisher’s exact test; 1 of 3 male patients had benign lesions), laterality (*p* = 0.248), or lesion morphology (*p* = 0.213). Detailed characteristics are summarized in **Table 1**. Inter-reader agreement for the Kaiser score was excellent (ICC = 0.911, 95% CI: 0.886-0.931; **Supplementary Table 2**). Although individual ADC measurements were not recorded separately, ADC inter-reader reproducibility in breast MRI has been established as excellent in prior multicenter studies (interreader ICC = 0.92; 95% CI: 0.80–0.97) (17).

**Table 1:** Clinical characteristics and pathological distribution of patients.

Characteristic	Total (n=222)	Benign (n=121)	Malignant (n=101)	p-value
Patient Characteristics				
Age, years (mean ± SD)	47.6 ± 11.1	44.5 ± 9.7	51.4 ± 11.6	<0.001
Female gender, n (%)	219 (98.6)	120 (99.2)	99 (98.0)	0.592
Male gender	3 (1.35)	1 (0.8)	2 (2)	
Lesion Characteristics Laterality, n (%)				0.248
Left breast	128 (57.7)	74 (61.2)	54 (53.5)	
Right breast	94 (42.3)	47 (38.8)	47 (46.5)	
Lesion morphology, n (%)				0.213
Mass	165 (74.3)	94 (77.7)	71 (70.3)	
Non-mass enhancement	57 (25.7)	27 (22.3)	30 (29.7)	
Pathological Diagnosis, n (%)				
Benign lesions	121 (54.5)			
Fibroadenoma	54 (24.3)	54 (44.6)	–	
Breast hyperplasia	39 (17.6)	39 (32.2)	–	
Intraductal papilloma	20 (9.0)	20 (16.5)	–	
Other benign lesions	8 (3.6)	8 (6.6)	–	
Malignant lesions	101 (45.5)			
Invasive ductal carcinoma	58 (26.1)	–	58 (57.4)	
Ductal carcinoma in situ	21 (9.5)	–	21 (20.8)	
Mucinous carcinoma	6 (2.7)	–	6 (5.9)	
Other malignant lesions	16 (7.2)	–	16 (15.8)	

Data are presented as mean ± SD or n (%). P-values compare benign vs malignant groups using independent t-test for age, Fisher’s exact test for gender (due to small, expected cell counts), and χ² test for laterality and lesion morphology. For pathological diagnosis, percentages in Total column are relative to all 222 lesions; percentages in Benign and Malignant columns are within each subgroup.

### 3.2 Diagnostic performance of Kaiser score, Kaiser+ score, and ADC model

**Figure 3:**
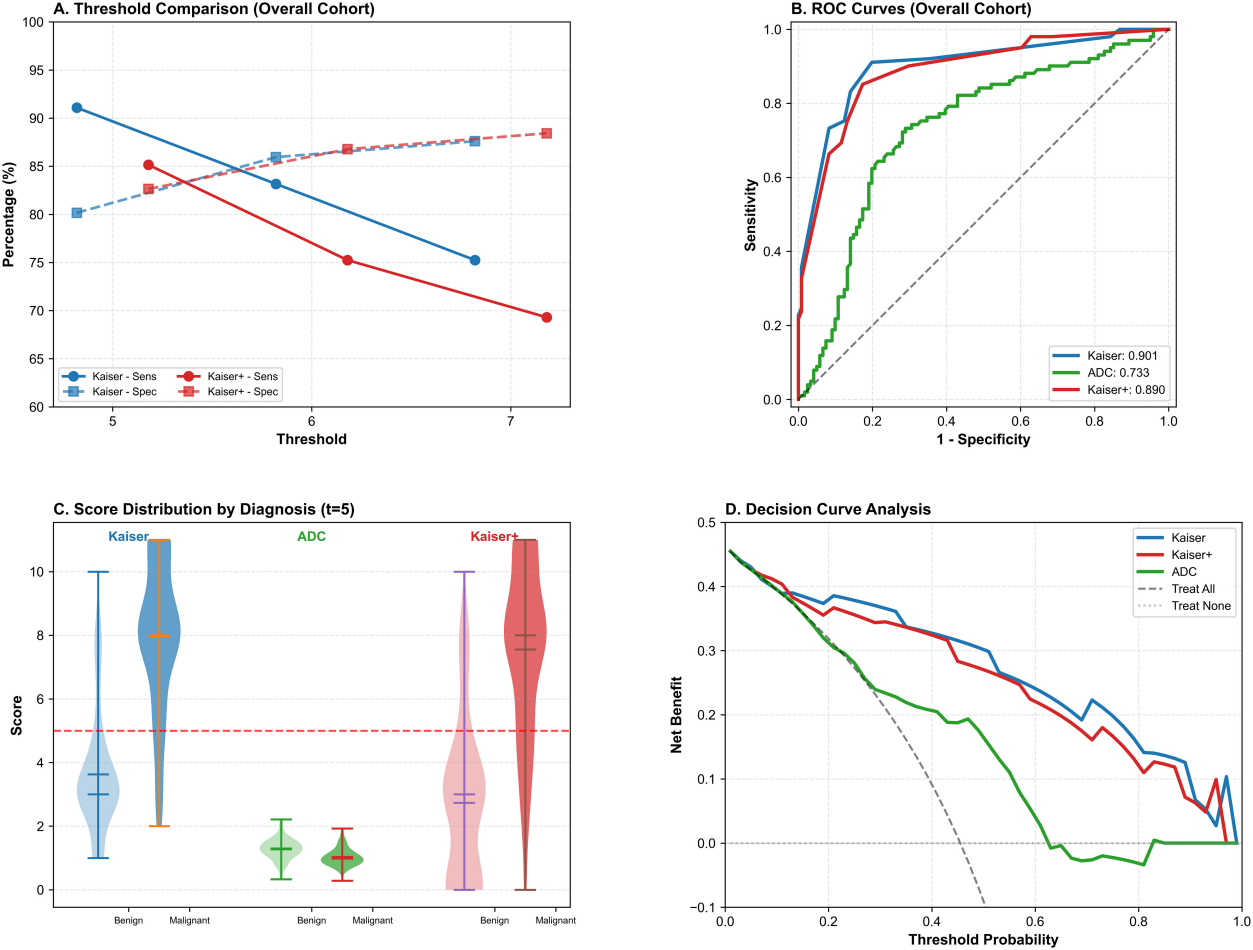
Threshold behavior, ROC performance, score distributions, and decision-curve analysis of the three diagnostic models in the overall cohort. **(A)** Sensitivity–specificity trade-off for the Kaiser and Kaiser+ models across thresholds 5–7; the study operating point is t = 5. **(B)** ROC curves (overall cohort) with AUCs: Kaiser 0.901 (95% CI: 0.856–0.941), Kaiser+ 0.890 (95% CI: 0.845–0.930), ADC 0.733 (95% CI: 0.666–0.800). **(C)** Score distributions by final diagnosis for the three models at t = 5: Kaiser scores, ADC values (×10^-3^ mm²/s, displayed as ×10 on axis), and Kaiser+ scores; red dashed line marks the ≥5 decision threshold for Kaiser/Kaiser+ models. **(D)** Decision-curve analysis (overall cohort) comparing net benefit across threshold probabilities; the Kaiser and Kaiser+ models yielded higher net benefit than treat-all and treat-none strategies across threshold probabilities 0.10–0.50, with the Kaiser model achieving the highest net benefit overall. Notes: Analyses are per lesion with paired model comparisons. Lesions with ADC ≤ 1.4 × 10^-3^ mm²/s were classified as malignant by the ADC rule. Kaiser+ = Kaiser score − 4 points when ADC > 1.4 × 10^-3^ mm²/s, otherwise unchanged; malignancy threshold for Kaiser/Kaiser+ defined as score ≥ 5. ADC, apparent diffusion coefficient; ROC, receiver operating characteristic; AUC, area under the curve; CI, confidence interval.

Using a threshold of ≥5 for Kaiser-based models and ≤1.4 × 10^-3^ mm²/s for ADC, the diagnostic performance of all three models was evaluated across the entire cohort and stratified by lesion morphology (**Table 2**; **Supplementary Table 3**–**5**; **Figure 3**).

**Table 2:** Diagnostic performance by lesion type.

Lesion type	Model	AUC (95% CI)	TP	FP	FN	TN	Sensitivity (%)	Specificity (%)	PLR (95% CI)	NLR (95% CI)	Accuracy (%)
Overall	Kaiser	0.901 (0.856-0.941)	92	24	9	97	91.1 (83.9-95.2)	80.2 (72.2-86.3)	4.59 (3.19-6.60)	0.111 (0.059-0.209)	85.1
Overall	Kaiser+	0.890 (0.845-0.930)	86	21	15	100	85.1 (76.9-90.8)	82.6 (74.9-88.4)	4.91 (3.30-7.30)	0.180 (0.112-0.289)	83.8
Overall	ADC	0.733 (0.666-0.800)	90	80	11	41	89.1 (81.5-93.8)	33.9 (26.1-42.7)	1.35 (1.17-1.56)	0.321 (0.174-0.592)	59.0
Mass	Kaiser	0.926 (0.878-0.965)	65	14	6	80	91.5 (82.8–96.1)	85.1 (76.5–90.9)	6.15 (3.77–10.02)	0.099 (0.046–0.215)	87.9
Mass	Kaiser+	0.909 (0.861-0.954)	60	13	11	81	84.5 (74.3–91.1)	86.2 (77.8–91.7)	6.11 (3.65–10.22)	0.180 (0.104–0.311)	85.5
Mass	ADC	0.749 (0.665-0.827)	61	60	10	34	85.9 (76.0–92.2)	36.2 (27.2–46.2)	1.35 (1.13–1.61)	0.389 (0.207–0.734)	57.6
Non-mass	Kaiser	0.801 (0.681-0.905)	27	10	3	17	90.0 (74.4–96.5)	63.0 (44.2–78.5)	2.43 (1.46–4.03)	0.159 (0.052–0.483)	77.2
Non-mass	Kaiser+	0.809 (0.690-0.910)	26	8	4	19	86.7 (70.3–94.7)	70.4 (51.5–84.1)	2.93 (1.61–5.32)	0.189 (0.074–0.487)	78.9
Non-mass	ADC	0.663 (0.518-0.802)	29	20	1	7	96.7 (83.3–99.4)	25.9 (13.2–44.7)	1.31 (1.03–1.65)	0.129 (0.017–0.979)	63.2
P-value		0.002	–	–	–	–	0.842	<0.001	–	–	0.003

AUC, area under the receiver operating characteristic curve; PLR, positive likelihood ratio; NLR, negative likelihood ratio; TP, true positive; FP, false positive; FN, false negative; TN, true negative.ADC, apparent diffusion coefficient. P-values comparing mass vs non-mass lesion groups. DeLong test for AUC comparison; chi-square test for other metrics. Kaiser score and Kaiser+ score used threshold of 5 (scores >5 considered malignant); ADC used threshold of 1.4 × 10^-3^ mm²/s (values <1.4 considered malignant).

#### 3.2.1 Overall diagnostic performance (n=222)

The Kaiser score demonstrated excellent diagnostic performance, achieving an AUC of 0.901 (95% CI: 0.856–0.941), with a sensitivity of 91.1%, specificity of 80.2%, and overall accuracy of 85.1%. The Kaiser+ model demonstrated comparable overall performance (AUC 0.890; 95% CI: 0.845–0.930), with slightly lower sensitivity (85.1%) but marginally improved specificity (82.6%) and similar accuracy (83.8%). The difference in AUC between the Kaiser and Kaiser+ models was not statistically significant [ΔAUC = 0.011; *p* = 0.622 (raw), *p* = 1.000(adj); DeLong test with Bonferroni correction]. In marked contrast, the ADC model demonstrated significantly inferior performance (AUC 0.733; 95% CI: 0.666–0.800), with significantly lower AUC compared with both the Kaiser model [ΔAUC = 0.168; *p* < 0.001(raw), *p* < 0.001(adj)] and the Kaiser+ model [ΔAUC = 0.157; *p* < 0.001(raw), *p* < 0.001(adj); DeLong test with Bonferroni correction]. Despite high sensitivity (89.1%), the ADC model exhibited markedly low specificity (33.9%), resulting in poor overall accuracy (59.0%) and a low positive likelihood ratio (PLR = 1.35; 95% CI: 1.17–1.56), which indicates limited clinical utility. Decision curve analysis showed Kaiser and Kaiser+ yielded higher net benefit than ADC across threshold probabilities (0.10–0.50). At a threshold probability of 0.20, the Kaiser model achieved a net benefit of 0.371 compared with 0.319 for the treat-all strategy (ΔNB = 0.052). The net benefit of the ADC model decreased to ≤0 at threshold probabilities ≥0.63, suggesting potential net harm relative to a treat-none strategy (**Supplementary Table 4**; **Figure 3**).

#### 3.2.2 Performance by lesion morphology

**Figure 4:**
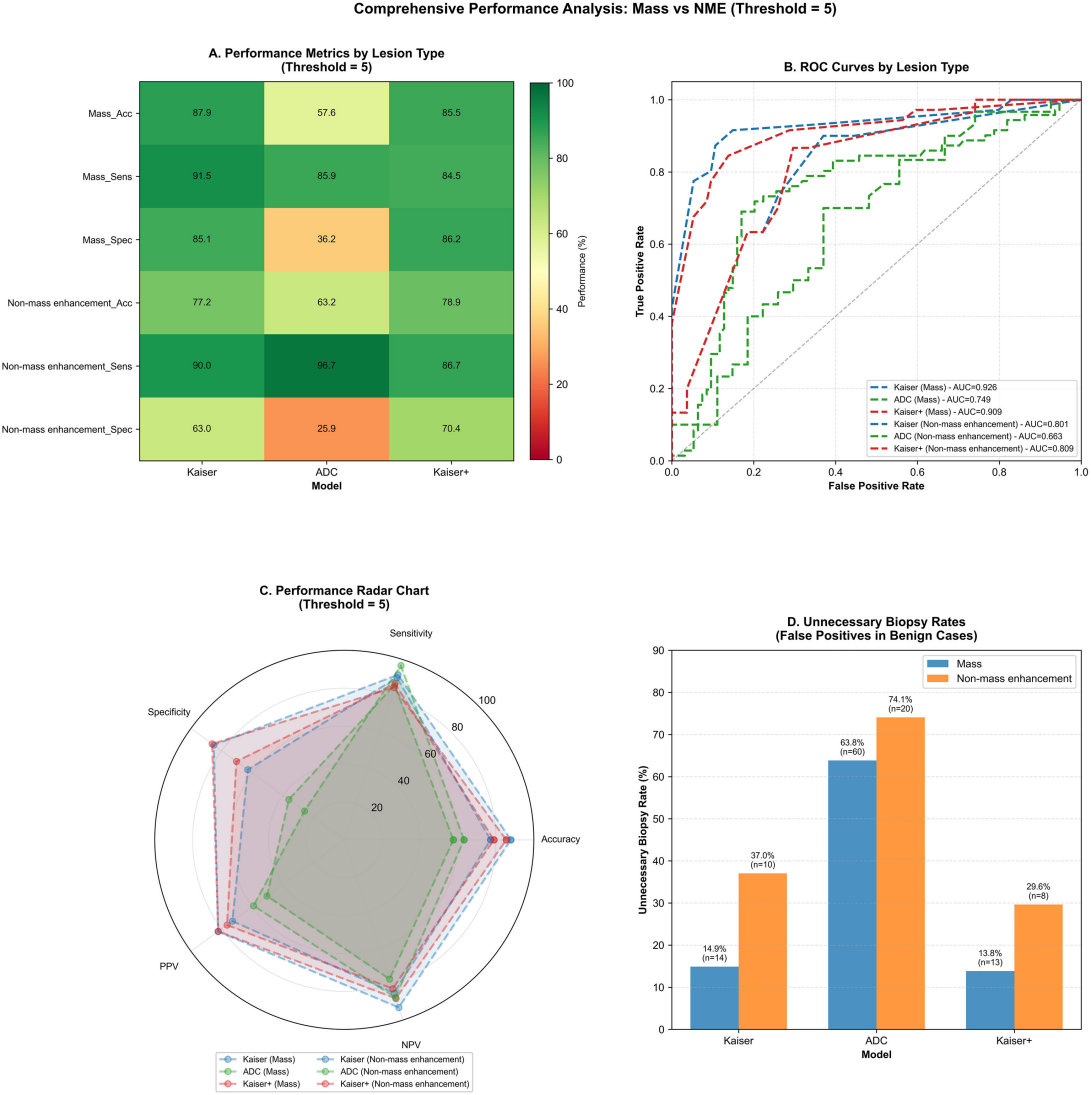
Comprehensive performance analysis: mass vs. non-mass enhancement lesions (Threshold ≥5) **(A)** Performance metrics heatmap by lesion type (Mass and Non-mass enhancement). **(B)** ROC curves comparing AUC across models and lesion types (solid lines = Mass; dashed lines = Non-mass enhancement). **(C)** Radar chart displaying five diagnostic metrics (accuracy, sensitivity, specificity, PPV, and NPV) for each model by lesion type. **(D)** Unnecessary biopsy rates (FP rates among benign lesions) stratified by lesion morphology. The Kaiser and Kaiser+ models demonstrated superior diagnostic performance compared with the ADC model in both mass and NME lesions. Sample sizes: overall n = 222 (121 benign, 101 malignant), mass n = 165 (94 benign, 71 malignant), NME n = 57 (27 benign, 30 malignant). Diagnostic thresholds: Kaiser/Kaiser+ ≥5; ADC ≤1.4 × 10^-3^ mm²/s.

The Kaiser model demonstrated superior diagnostic performance in mass lesions (n = 165) compared with NME lesions (n = 57). For mass lesions, the Kaiser model achieved an AUC of 0.926 (95% CI: 0.878–0.965), with a sensitivity of 91.5%, specificity of 85.1%, and accuracy of 87.9%. In NME lesions, performance decreased to an AUC of 0.801 (95% CI: 0.681–0.905), with a sensitivity of 90.0%, specificity of 63.0%, and accuracy of 77.2%. The Kaiser+ model demonstrated similar patterns, achieving AUC values of 0.909 (95% CI: 0.861–0.954) for mass lesions and 0.809 (95% CI: 0.690–0.910) for NME lesions. The ADC model exhibited consistently poor specificity across both morphologic subtypes (36.2% for mass lesions and 25.9% for NME lesions), resulting in low overall accuracy (57.6% and 63.2%, respectively), despite maintaining high sensitivity in both groups. This pattern was consistent across both morphologic subtypes: the Kaiser and Kaiser+ models remained significantly superior to the ADC model after Bonferroni correction, while the difference between Kaiser and Kaiser+ remained non-significant [all *p* ≥ 0.05(adj)]. Detailed results are presented in **Table 2**, **Supplementary Table 3**, **Figure 4**.

#### 3.2.3 Clinical implications for biopsy decision-making

The confusion matrix data and potential biopsy avoidance rates are detailed in **Supplementary Table 6**. Applying the Kaiser score at a threshold of ≥5, 97 of 121 benign lesions (80.2%) could potentially have avoided unnecessary biopsy, corresponding to 24 FP cases. The biopsy avoidance rate was higher for mass lesions (85.1%; 80 of 94 benign lesions) compared with NME lesions (63.0%; 17 of 27 benign lesions). Regarding FN rates, the Kaiser model missed 9 malignant lesions (FN rate: 8.9%), the Kaiser+ model missed 15 (14.9%), and the ADC model missed 11 (10.9%). The Kaiser+ model showed marginally improved biopsy avoidance (82.6%; 100 of 121 benign lesions) but at the cost of a higher FN rate (14.9% vs. 8.9% for the Kaiser model). In contrast, the ADC model produced a substantially higher FP burden (80/121 = 66.1% of benign lesions), indicating limited clinical utility in this cohort.

### 3.3 Diagnostic errors by pathological subtype

**Figure 5:**
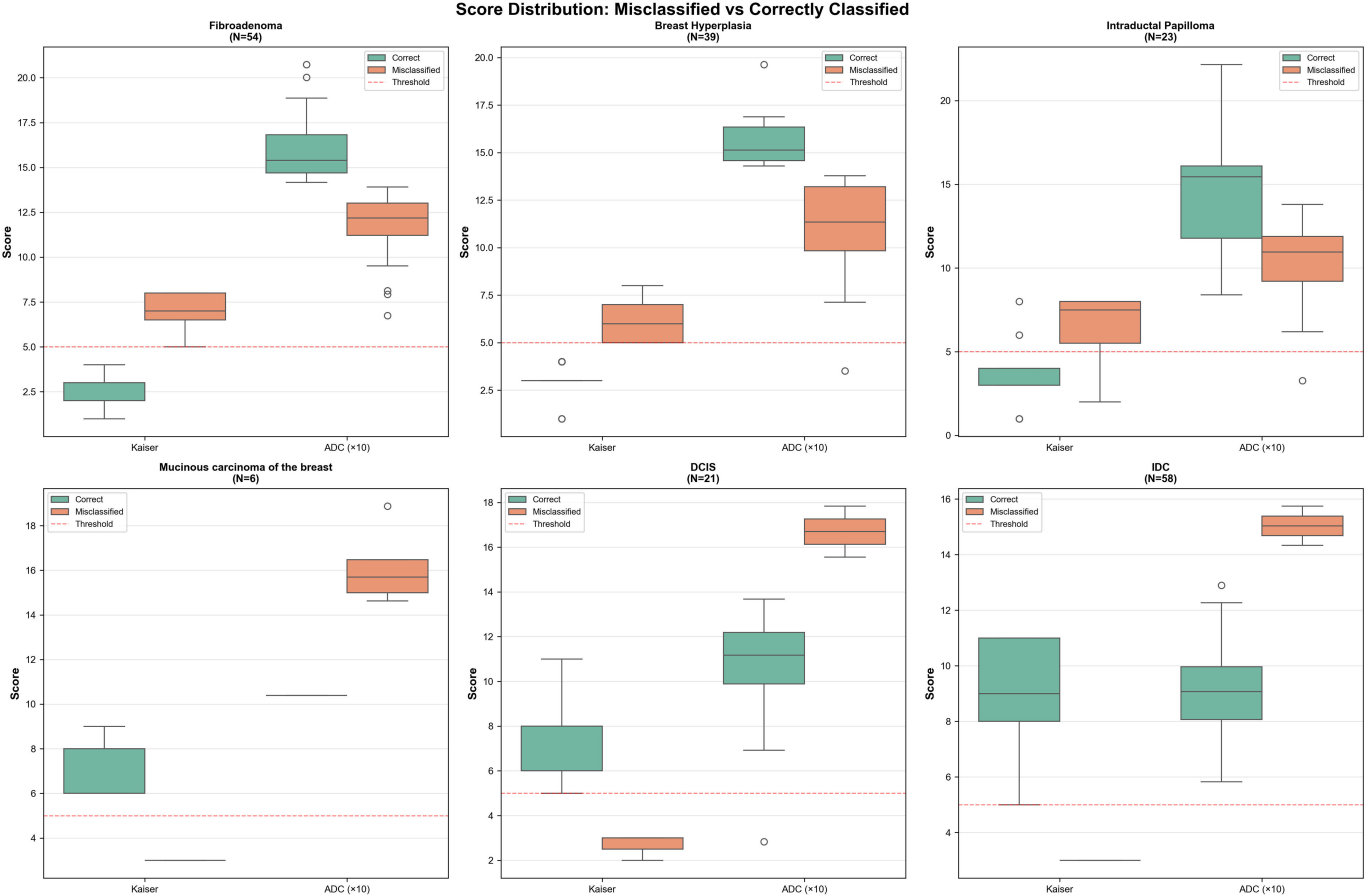
Score distributions by pathology subtype: correctly vs misclassified at t=5. fibroadenoma (N = 54), breast hyperplasia (N = 39), intraductal papilloma (N = 23, comprising 20 benign and 3 malignant cases; the 3 malignant cases were included in ‘other malignant lesions’ in **Table 1**), mucinous carcinoma (N = 6), DCIS (N = 21), IDC (N = 58). Boxplots show Kaiser scores and ADC values (×10) split by correct vs misclassified decisions; red dashed line marks the Kaiser/Kaiser+ threshold = 5 (applies to score panels only). Misclassified = FP for benign or FN for malignant, against histopathology. Boxes, IQR with median; whiskers, 1.5×IQR; dots, outliers. (ADC rule uses cut-off ≤1.4×10^-3^ mm²/s ≈ 14 on the plotted scale.).

Among the 222 lesions, 33 (14.9%) were misclassified by the Kaiser model, 36 (16.2%) by the Kaiser+ model, and 91 (41.0%) by the ADC model. Subtype-specific analyses distinguished between FN errors (missed malignancies) and FP errors (benign lesions incorrectly classified as malignant) across all three models. All pairwise comparisons of FP and FN rates were performed using McNemar’s test with Bonferroni correction, with benign and malignant lesions analyzed separately (adjusted α = 0.017 for three pairwise comparisons) (**Table 3**; **Supplementary Table 7**; **Figure 5**).

**Table 3:** Misclassification summary at the fixed operating point (t = 5).

A. Overall misclassification (n = 222)				
Model	Misclassified, n (%)			Components (FN + FP)
Kaiser	33 (14.9%)			9 + 24
Kaiser+	36 (16.2%)			15 + 21
ADC	91 (41.0%)			11 + 80
B. Malignant lesions: false-negative (FN) rates (n = 101)				
Subtype (n)	Kaiser, n/N (%) [95% CI]	Kaiser+, n/N (%) [95% CI]	ADC, n/N (%) [95% CI]	Pairwise (McNemar, Bonferroni-adjusted)
All malignant (101)	9/101 (8.9%) [4.8–16.1]	15/101 (14.9%) [9.2–23.1]	11/101 (10.9%) [6.2–18.5]	K vs ADC: p_raw≥0.333, *p* ≥0.999[adj]; K+ vs ADC: *p*≥0.333[raw], *p*≥0.999[adj]; K vs K+: *p* = 0.041[raw], *p* = 0.123[adj]
IDC (58)	1/58 (1.7%)	2/58 (3.4%)	2/58 (3.4%)	–
DCIS (21)	3/21 (14.3%)	4/21 (19.0%)	2/21 (9.5%)	–
Mucinous carcinoma (6)	1/6 (16.7%)	4/6 (66.7%)	5/6 (83.3%)	–
C. Benign lesions: false-positive (FP) rates (n = 121)				
Subtype (n)	Kaiser, n/N (%) [95% CI]	Kaiser+, n/N (%) [95% CI]	ADC, n/N (%) [95% CI]	Pairwise (McNemar, Bonferroni-adjusted)
All benign (121)	24/121 (19.8%) [13.7–27.8]	21/121 (17.4%) [11.6–25.1]	80/121 (66.1%) [57.3–73.9]	K vs ADC: *p* < 0.001[raw], *p* < 0.001[adj]; K+ vs ADC: *p* < 0.001[raw], *p* < 0.001[adj]; K vs K+: *p* = 0.248[raw], *p* = 0.744[adj]
Fibroadenoma (54)	8/54 (14.8%) [7.7–26.6]	8/54 (14.8%) [7.7–26.6]	32/54 (59.3%) [46.0–71.3]	-
Breast hyperplasia (39)	9/39 (23.1%) [12.6–38.3]	7/39 (17.9%) [9.0–32.7]	27/39 (69.2%) [53.6–81.4]	-
Intraductal papilloma (20)	5/20 (25.0%) [11.2–46.9]	4/20 (20.0%) [8.1–41.6]	14/20 (70.0%) [48.1–85.5]	-

Values are n/N (%) with 95% CI (Wilson method). Threshold fixed at t, 5. McNemar tests used Bonferroni correction; both raw and Bonferroni-adjusted p-values are reported; false-positive and false-negative rates analyzed separately, adjusted α, 0.017. IDC, invasive ductal carcinoma; DCIS, ductal carcinoma in situ; Kaiser, Kaiser score; K+, Kaiser+; FN, false-negative; FP, false-positive.

FN rates were 8.9% (9/101; 95% CI: 4.8–16.1) for the Kaiser model, 14.9% (15/101; 95% CI: 9.2–23.1) for the Kaiser+ model, and 10.9% (11/101; 95% CI: 6.2–18.5) for the ADC model. Kaiser vs. ADC: *p*≥ 0.333[raw], *p* ≥ 0.999[adj]; Kaiser+ vs. ADC: *p* ≥ 0.333[raw], *p* ≥ 0.999[adj]; Kaiser vs. Kaiser+: *p* = 0.041[raw], *p* = 0.123[adj]. By subtype, IDC (n = 58) showed very low FN rates across all models [Kaiser 1.7% (1/58); Kaiser+ 3.4% (2/58); ADC 3.4% (2/58)]; DCIS (n = 21) showed similar rates [Kaiser 14.3% (3/21); Kaiser+ 19.0% (4/21); ADC 9.5% (2/21)]. Mucinous carcinoma (n = 6) showed substantially elevated FN rates [Kaiser: 16.7% (1/6); Kaiser+: 66.7% (4/6); ADC: 83.3% (5/6)]; however, these findings should be interpreted with caution given the very small sample size and are considered exploratory only.

In benign lesions (n = 121), the ADC model produced 80 FP cases (66.1%; 95% CI: 57.3–73.9), substantially exceeding the Kaiser model [24/121;19.8%; 95% CI: 13.7–27.8; *p* < 0.001 (raw), *p* < 0.001 (adj)] and the Kaiser+ model [21/121;17.4%;95% CI: 11.6–25.1; *p* < 0.001 (raw), *p* < 0.001(adj)], while the Kaiser vs. Kaiser+ comparison showed no significant FP difference (*p* = 0.248 [raw], *p* = 0.744 [adj]). By subtype, fibroadenoma (n = 54) showed FP rates of 14.8% (8/54; 95% CI: 7.7–26.6) for both the Kaiser and Kaiser+ models, compared with 59.3% (32/54; 95% CI: 46.0–71.3) for the ADC model. Breast hyperplasia (n = 39) showed a similar pattern [Kaiser 23.1%: (9/39; 95% CI: 12.6–38.3); Kaiser+ 17.9% (7/39; 95% CI: 9.0–32.7); ADC 69.2% (27/39;95% CI: 53.6–81.4)]. Intraductal papilloma (n = 20, comprising 20 benign and 3 malignant cases; the 3 malignant cases were included under ‘other malignant lesions’ in **Table 1**) also exhibited high FP rates across all models (Kaiser: 25.0% [5/20; 95% CI: 11.2–46.9]; Kaiser+: 20.0% [4/20; 95% CI: 8.1–41.6]; ADC: 70.0% [14/20; 95% CI: 48.1–85.5]).

## 4 Discussion

Across the entire cohort, the Kaiser model demonstrated the highest diagnostic performance, followed by the Kaiser+ and ADC models, consistent with the overall AUC ranking reported in **Table 2**. The Kaiser model showed significantly superior AUC compared with the ADC model, whereas no significant difference in AUC was observed between the Kaiser and Kaiser+ models (**Table 2**; **Supplementary Table 3**; **Figure 3**). These findings are consistent with prior studies. Meng et al. (10) and An et al. (20) reported no significant AUC improvement when ADC was combined with the Kaiser score. Pan et al. (21) similarly observed no additional diagnostic benefit from ADC integration. In contrast, Chen et al. (22) demonstrated that Kaiser+ achieved significantly improved specificity compared with Kaiser alone (68.5% vs. 59.1%; *p* < 0.0001), albeit with a modest reduction in sensitivity and no significant AUC gain, suggesting that ADC integration selectively improves specificity rather than overall discriminatory power. Across these studies, lower diagnostic accuracy was consistently observed for NME lesions compared with mass lesions across all scoring systems, reinforcing the inherent challenge of characterizing NME regardless of whether morphologic or diffusion parameters are used. For mass lesions, this may be explained by the fact that diffusion features often correlate with malignant morphologic cues already captured by the Kaiser score. The excellent inter-reader agreement for Kaiser scoring (ICC = 0.911; 95% CI: 0.886–0.931) supports the reproducibility of this structured assessment tool among experienced readers, consistent with prior validation studies reporting ICC values of 0.852–0.882 (22, 23). This level of agreement is clinically meaningful, suggesting that Kaiser score-based decisions are likely to be consistent across readers with comparable experience, supporting its potential for broader clinical implementation.

Beyond overall diagnostic metrics, the present study examined misclassification patterns from a pathology-driven perspective. Diagnostic errors were not randomly distributed but clustered within histologic subtypes exhibiting inherent imaging–pathology discordance (**Table 3**; **Figure 5**). Three benign cellular or proliferative entities—fibroadenomas, breast hyperplasia, and intraductal papillomas—accounted for the majority of FP cases across all models. Among malignant lesions, low-conspicuity subtypes—specifically DCIS and mucinous carcinoma—were associated with the majority of FN errors. Notably, paired testing revealed that the performance gap between models was primarily attributable to differences in specificity for benign lesions rather than sensitivity for malignant lesions.

These entities share hormone-responsive vascularity and epithelial proliferation yet produce distinct imaging signatures that lead to model-specific diagnostic errors. At the cohort level, the Kaiser model maintained a substantially lower FP rate than the ADC model, with the Kaiser+ model demonstrating only marginal additional improvement in specificity. Fibroadenomas may develop lobulated contours and exhibit brisk enhancement (24–26), thereby elevating Kaiser scores despite benign histology; cellular stroma composition also lowers ADC to malignant-like ranges (27). Consequently, at the prespecified ADC threshold of 1.4 × 10^-3^ mm²/s, a substantial proportion of fibroadenomas were incorrectly classified as malignant, yielding a markedly higher FP rate for the ADC model than for the Kaiser and Kaiser+ models. Breast hyperplasia (n = 39) manifests as regional NME with elevated Kaiser scores (28), and increased epithelial cellularity contributes to reduced ADC values (7), resulting in the highest FP rate among all models for ADC, while the Kaiser and Kaiser+ models performed comparably. Intraductal papillomas (n = 20) produce focal intraductal enhancement that elevates morphology-based scores (29), while epithelial compactness lowers ADC values (30), similarly resulting in a substantially higher FP rate for the ADC model compared with the Kaiser and Kaiser+ models. The morphology–kinetic integration inherent to the Kaiser score mitigates diffusion overlap in cellular benign lesions, explaining its substantially lower FP rate compared with ADC alone, whereas the incremental benefit of ADC integration (Kaiser+) for these benign phenotypes remains modest.

Among malignant subtypes, IDC(n = 58) typically exhibits desmoplasia and neo-angiogenesis, manifesting as irregular margins, internal enhancement heterogeneity, and washout kinetics, thereby raising multiple Kaiser score criteria (31, 32). Correspondingly, FN rates were low across all models: 1.7% (1/58) for Kaiser, 3.4% (2/58) for Kaiser+, and 3.4% (2/58) for ADC. In contrast, DCIS (n=21), Confined to the ducts without stromal invasion, lacks the desmoplastic–angiogenic cascade of invasive disease and more often shows plateau or persistent kinetics with smooth margins (31, 33). ADC showed comparable limitations as intraductal epithelial proliferation without stromal desmoplasia yields intermediate diffusion values that overlap with those of benign NME (34–36). FN rates were 14.3% (3/21) for Kaiser, 19.0% (4/21) for Kaiser+, and 9.5% (2/21) for ADC. Mucinous carcinoma (n = 6) presents unique diagnostic challenges, as abundant extracellular mucin (≈50–90% of tumor volume) produces markedly elevated T2 signal, elevated ADC (~1.2–2.3 × 10^-3^ mm²/s), and smooth, lobulated contours (6, 36). All three models demonstrated limited diagnostic performance: Kaiser yielded low scores for these lesions due to low kinetic suspicion and rounded morphology, while ADC misclassified lesions as benign due to high diffusion. FN rates were 16.7% (1/6) for Kaiser, 66.7% (4/6) for Kaiser+, and 83.3% (5/6) for ADC. Given the very small sample size (n=6), these subtype-specific findings should be regarded as exploratory and hypothesis-generating and require confirmation in larger dedicated cohorts before clinical recommendations can be made.

At the cohort level, the Kaiser model substantially reduced benign FPs compared with the ADC model; the Kaiser+ model further reduced FPs, albeit without a significant gain in overall AUC. This pattern is consistent with partial information redundancy between morphokinetic enhancement heterogeneity and restricted diffusion characteristics in cellular lesions, whereby ADC provides limited incremental discriminatory value beyond what is already captured by the Kaiser score.

For clinical implementation, a Kaiser-first pathway is recommended, with ADC serving as a secondary discriminator for lesions with intermediate Kaiser scores (5–7). Future studies should evaluate subtype-aware ADC thresholds and probabilistic frameworks that incorporate ADC as a likelihood modifier rather than a fixed additive adjustment, particularly for low-conspicuity malignancies such as DCIS and mucinous carcinoma, where morphokinetic features alone may be insufficient. Importantly, the presence of FN cases for DCIS and mucinous carcinoma underscores a critical clinical caveat: the Kaiser score should not be used as a standalone tool to exclude biopsy in all BI-RADS 4 lesions. When imaging features raise suspicion for DCIS (e.g., NME with plateau or persistent kinetics) or mucinous carcinoma (e.g., markedly elevated T2 signal intensity, smooth lobulated contours, and elevated ADC values), biopsy should be strongly considered regardless of the Kaiser score or ADC threshold.

This study has several limitations. First, this was a single-center retrospective analysis spanning 2014–2024, conducted using mixed 1.5-T and 3.0-T MRI systems from a single vendor. Detailed per-scanner logs were incomplete, and no field–strength–specific harmonization or cohort-specific optimization of the ADC decision rule (1.4 × 10^-3^ mm²/s, adopted from Baltzer et al. (18)) was performed. Second, pathology subtypes were imbalanced, and some subgroups were small (e.g., mucinous carcinoma, n = 6; DCIS subgroups), which reduces power and widens CIs for subgroup and misclassification analyses despite paired testing. Third, diffusion data were derived from a single-shell acquisition (b=0 and 800 s/mm²); more advanced models (e.g., intravoxel incoherent motion [IVIM], diffusion tensor imaging [DTI]) and standardized post-processing may better capture tissue microstructure and could be explored prospectively. Fourth, the primary reading used consensus scores; although inter-reader agreement was quantified, consensus may inflate apparent performance relative to fully independent reads. Fifth, the Kaiser+ model used a fixed categorical adjustment (−4 points when ADC exceeded the threshold) rather than a data-driven integration; calibration and model optimization were beyond the scope of this study, and alternative approaches may yield different decision boundaries. Furthermore, the Kaiser+ model in this study represents a purely MRI-based integration of the Kaiser score and ADC, without incorporation of mammographic features such as microcalcifications; this should be considered when comparing results with multimodality approaches. Sixth, the cohort comprised biopsy-enriched BI-RADS 4 lesions from a single institution, which limits generalizability of prevalence-dependent metrics and risks spectrum or verification bias; accordingly, PPV and NPV were not reported, and PLR and NLR were used instead as prevalence-independent measures of diagnostic performance. Seventh, only one lesion per patient was included to avoid clustering effects; however, this approach may have excluded multifocal small lesions that are common in clinical practice and are inherently more challenging to evaluate using the Kaiser score and ADC, potentially inflating diagnostic performance metrics and limiting generalizability to real-world scenarios involving multiple simultaneous BI-RADS 4 lesions. Eighth, the high exclusion rate (~96.4%; 222 of 6, 129 screened patients were ultimately included) introduces potential selection bias. Common exclusion reasons—such as poor image quality or absence of histopathologic confirmation—systematically favor larger, higher-quality lesions that are easier to assess with the Kaiser score and ADC, potentially excluding the most diagnostically challenging cases that drive clinical uncertainty and thereby inflating reported performance metrics. Finally, external, multicenter validation with harmonized protocols and independent readers is required to confirm robustness across vendors, field strengths, and practice settings.

## 5 Conclusion

In BI-RADS 4 lesions, the Kaiser score demonstrated superior diagnostic performance compared with ADC alone; the Kaiser+ did not significantly increase AUC but improved specificity in NME lesions. Pathology-stratified analysis revealed subtype-dependent misclassification patterns: benign cellular and proliferative lesions—fibroadenoma, breast hyperplasia, and intraductal papilloma—accounted for the majority of FP errors, with substantially higher rates observed under the ADC model than under Kaiser-based models. Conversely, DCIS and mucinous carcinoma accounted for the majority of FN errors across all models, attributable to their atypical morphokinetic and diffusion characteristics. Awareness of these subtype-specific diagnostic pitfalls may assist radiologists in interpreting borderline BI-RADS 4 lesions with greater caution and support more individualized, evidence-based biopsy decision-making in clinical practice.

## Data Availability

The raw data supporting the conclusions of this article will be made available by the authors, without undue reservation.
