# Supplementary material for: Diagnostic performance of Kaiser score, ADC, and their combination for BI-RADS 4 breast lesions: subgroup and misclassification analysis
Source: Front Oncol. 2026 Jul 23;16:1802360. doi: 10.3389/fonc.2026.1802360 (PMC13441916; doi:10.3389/fonc.2026.1802360)
Supplement: Supplementary file 1 [file Table1.docx]

**Supplementary Table Legend**

**Supplementary Table 1. MRI Scan Parameters**

| **Sequence** | **TR (ms)** | **TE (ms)** | **Slice thickness (mm)** | **FOV (mm)** | **b-value (s/mm²)** |
| --- | --- | --- | --- | --- | --- |
| **1.5T Avanto** |  |  |  |  |  |
| T1WI/TSE | 8.6 | 4.7 | 1.2 | 340 × 100 | - |
| T2WI/TSE | 4640 | 69 | 3.0 | 370 × 100 | - |
| DWI | 6500 | 108 | 4.0 | 340 × 50 | 800 |
| dyn-VIEWS (CE-MRI) | 4.43 | 1.73 | 1.7 | 340 × 100 | - |
| **3.0T Skyra** |  |  |  |  |  |
| T1WI/TSE | 6.04 | 2.46 | 1.6 | 360 × 100 | - |
| T2WI/TSE | 5700 | 84 | 4.0 | 400 × 100 | - |
| DWI | 5300 | 58 | 4.0 | 340 × 60 | 800 |
| dyn-VIEWS (CE-MRI) | 4.31 | 1.61 | 1.5 | 360 × 100 | - |

MRI scan parameters for 1.5T Avanto and 3.0T Skyra scanners, including T1WI, T2WI, DWI sequences, and dynamic contrast-enhanced imaging (dyn-VIEWS). The contrast agent (Gd-DTPA) was administered intravenously at 0.2 ml/kg with a flow rate of 2 ml/s, followed by a 20 ml saline flush. Five dynamic post-contrast phases were acquired with ~54-second intervals.

**Supplement Table 2 Inter-rater Agreement for Kaiser Score Assessment**

| Assessment Parameter | ICC (2,1) | 95% CI | Agreement Level |
| --- | --- | --- | --- |
| Kaiser Score | 0.911 | 0.886-0.931 | Excellent |

Inter-rater reliability was assessed using the Intraclass Correlation Coefficient (ICC) with a two-way random effects model for absolute agreement [ICC (2,1)]. Both readers independently evaluated all 222 lesions prior to consensus discussion. ICC values were interpreted as follows: <0.50 = poor, 0.50-0.75 = moderate, 0.75-0.90 = good, and >0.90 = excellent agreement. The excellent ICC (0.911) demonstrates high reproducibility of the Kaiser score assessment between the two experienced breast radiologists.

**Supplement 3：Pairwise DeLong AUC comparisons among models within lesion groups (Overall, Mass, Non-mass–like) with Bonferroni correction**

| **Group** | **Comparison** | **AUC_Model1** | **AUC_Model2** | **Difference** | **Z_statistic** | **P_value** | **Significance** | **Bonferroni_Sig** |
| --- | --- | --- | --- | --- | --- | --- | --- | --- |
| Overall | Kaiser vs ADC | 0.901 | 0.733 | 0.168 | 5.59 | 0 | *** | *** |
| Overall | Kaiser vs Kaiser+ | 0.901 | 0.890 | 0.011 | 0.493 | 0.622 | NS | NS |
| Overall | Kaiser+ vs ADC | 0.890 | 0.733 | 0.157 | 5.179 | 0 | *** | *** |
| Mass | Kaiser vs ADC | 0.926 | 0.749 | 0.176 | 5.163 | 0 | *** | *** |
| Mass | Kaiser vs Kaiser+ | 0.926 | 0.909 | 0.017 | 0.689 | 0.491 | NS | NS |
| Mass | Kaiser+ vs ADC | 0.909 | 0.749 | 0.16 | 4.639 | 0 | *** | *** |
| NME | Kaiser vs ADC | 0.801 | 0.663 | 0.138 | 2.081 | 0.038 | * | * |
| NME | Kaiser vs Kaiser+ | 0.801 | 0.809 | -0.008 | -0.138 | 0.89 | NS | NS |
| NME | Kaiser+ vs ADC | 0.809 | 0.663 | 0.146 | 2.214 | 0.027 | * | * |

AUCs are taken from the main performance table for each model and lesion group. Pairwise AUC differences (ΔAUC) were tested using the **DeLong** method; **Bonferroni correction (m=3 per group: Kaiser vs ADC, Kaiser vs Kaiser+, Kaiser+ vs ADC)** was applied to control the family-wise error rate. Two-sided tests; α=0.05. Significance codes: NS (not significant), * P<0.05, ** P<0.01, *** P<0.001.

**Supplement 4：Decision curve analysis (overall cohort): net benefit across threshold probabilities for Kaiser, Kaiser+, and ADC**

| Threshold | Kaiser_NetBenefit | KaiserPlus_NetBenefit | ADC_NetBenefit | TreatAll_NetBenefit |
| --- | --- | --- | --- | --- |
| 0.01 | 0.45495495495495497 | 0.45495495495495497 | 0.45495495495495497 | 0.45495495495495497 |
| 0.02 | 0.4438315866887296 | 0.4438315866887296 | 0.4438315866887296 | 0.4438315866887296 |
| 0.03 | 0.44032692486300734 | 0.43809789170613916 | 0.43809789170613916 | 0.43809789170613916 |
| 0.04 | 0.43524774774774777 | 0.4322447447447448 | 0.4322447447447448 | 0.4322447447447448 |
| 0.05 | 0.4300616405879564 | 0.4262683736367947 | 0.4262683736367947 | 0.4262683736367947 |
| 0.06 | 0.41661874640598046 | 0.42208165612420934 | 0.4201648456967606 | 0.4201648456967606 |
| 0.07 | 0.4113629758791049 | 0.4178049016758694 | 0.41393005909134944 | 0.41393005909134944 |
| 0.08 | 0.40599294947121034 | 0.4161770466118292 | 0.4075597336466902 | 0.4075597336466902 |
| 0.09 | 0.4005049005049005 | 0.41208791208791207 | 0.4014949014949015 | 0.40104940104940107 |
| 0.1 | 0.3948948948948949 | 0.4079079079079079 | 0.39489489489489493 | 0.3943943943943944 |
| 0.11 | 0.3891588217430914 | 0.4036339710497014 | 0.3887033100516247 | 0.3875898370280393 |
| 0.12 | 0.3925061425061425 | 0.39926289926289926 | 0.3824733824733825 | 0.38063063063063063 |
| 0.13 | 0.38997618307963133 | 0.38329709019364194 | 0.37620378999689347 | 0.3735114424769597 |
| 0.14 | 0.38738738738738737 | 0.37890215797192545 | 0.3698931489629164 | 0.3662266918080872 |
| 0.15 | 0.3847376788553259 | 0.3744038155802862 | 0.35824059353471116 | 0.35877053524112346 |
| 0.16 | 0.382024882024882 | 0.36979836979836983 | 0.34727584727584726 | 0.3511368511368511 |
| 0.17 | 0.3792467165961142 | 0.36508194941929883 | 0.33984586996635185 | 0.3433192228372951 |
| 0.18 | 0.3764007910349374 | 0.3602504943968359 | 0.33223467369808835 | 0.33531092067677437 |
| 0.19 | 0.3734845957068179 | 0.3552997441886331 | 0.31993104215326434 | 0.32710488266043825 |
| 0.2 | 0.3704954954954955 | 0.35022522522522526 | 0.3130630630630631 | 0.3186936936936937 |
| 0.21 | 0.3856768160565629 | 0.3668035123731326 | 0.3049378492416467 | 0.31006956323412027 |
| 0.22 | 0.3839223839223839 | 0.36417186417186415 | 0.30168630168630167 | 0.3012243012243012 |
| 0.23 | 0.3821223821223821 | 0.36147186147186144 | 0.2961272961272961 | 0.2921492921492922 |
| 0.24 | 0.3802750118539592 | 0.3587008060692271 | 0.28473210052157416 | 0.2828354670459934 |
| 0.25 | 0.37837837837837834 | 0.35585585585585583 | 0.2807807807807808 | 0.27327327327327333 |
| 0.26 | 0.3764304845385926 | 0.3529340150961772 | 0.26941806671536406 | 0.26345264183102024 |
| 0.27 | 0.3744292237442922 | 0.3499321239047266 | 0.25681846229791433 | 0.253362951993089 |
| 0.28 | 0.37237237237237236 | 0.3468468468468468 | 0.24724724724724723 | 0.24299299299299298 |
| 0.29 | 0.3702575815251871 | 0.343674660576069 | 0.23962695089455652 | 0.23233092247176756 |
| 0.3 | 0.36808236808236805 | 0.34041184041184036 | 0.2368082368082368 | 0.22136422136422138 |
| 0.31 | 0.36584410497453973 | 0.34488836662749706 | 0.23384253819036424 | 0.21007964486225356 |
| 0.32 | 0.3635400105988341 | 0.34287228404875464 | 0.23158452570217278 | 0.19846316905140432 |
| 0.33 | 0.36116713728654026 | 0.34079601990049746 | 0.22791448164582492 | 0.18649993276858945 |
| 0.34 | 0.3587223587223587 | 0.3386568386568386 | 0.22440622440622437 | 0.17417417417417413 |
| 0.35000000000000003 | 0.3371448371448372 | 0.3364518364518364 | 0.2193347193347193 | 0.16146916146916146 |
| 0.36 | 0.33530405405405406 | 0.3341779279279279 | 0.2125563063063063 | 0.14836711711711714 |
| 0.37 | 0.3334048334048334 | 0.3318318318318318 | 0.21278421278421275 | 0.13484913484913486 |
| 0.38 | 0.33144434757337987 | 0.3294100552165068 | 0.2117117117117117 | 0.12089508863702414 |
| 0.39 | 0.3294195835179442 | 0.32690887608920394 | 0.20846256092157733 | 0.1064835327130409 |
| 0.4 | 0.32732732732732733 | 0.3243243243243243 | 0.20270270270270271 | 0.09159159159159158 |
| 0.41000000000000003 | 0.32516414719804554 | 0.32165216063521146 | 0.20484043365399296 | 0.07619483890670331 |
| 0.42 | 0.3229263746505126 | 0.318887853370612 | 0.19400434917676296 | 0.060267163715439664 |
| 0.43 | 0.32061008376797856 | 0.3160265528686581 | 0.1882408724513988 | 0.043780622727991214 |
| 0.44 | 0.31821106821106826 | 0.31306306306306303 | 0.19111969111969113 | 0.026705276705276726 |
| 0.45 | 0.31572481572481575 | 0.2833742833742834 | 0.18755118755118755 | 0.009009009009009084 |
| 0.46 | 0.31314647981314647 | 0.2809476142809476 | 0.19586252919586253 | -0.009342676 |
| 0.47000000000000003 | 0.3104708482066973 | 0.2784293727689954 | 0.1935237123916369 | -0.028386877 |
| 0.48 | 0.3076923076923077 | 0.2758142758142758 | 0.17359667359667358 | -0.048163548 |
| 0.49 | 0.3048048048048048 | 0.2730966260378025 | 0.17594064652888183 | -0.068715775 |
| 0.5 | 0.30180180180180183 | 0.2702702702702703 | 0.16216216216216217 | -0.09009009 |
| 0.51 | 0.29867622724765586 | 0.26732855304283876 | 0.15342893914322486 | -0.112336827 |
| 0.52 | 0.2691441441441441 | 0.26426426426426425 | 0.1445195195195195 | -0.135510511 |
| 0.53 | 0.2661491278512555 | 0.2610695802185164 | 0.13091815219474792 | -0.159670309 |
| 0.54 | 0.26302389345867605 | 0.25773599686643167 | 0.11868390129259694 | -0.184880533 |
| 0.55 | 0.25975975975975973 | 0.25425425425425424 | 0.11061061061061057 | -0.211211211 |
| 0.56 | 0.2563472563472563 | 0.2506142506142506 | 0.09500409500409498 | -0.238738739 |
| 0.5700000000000001 | 0.2527760318457993 | 0.24680494447936308 | 0.07867169495076469 | -0.267546616 |
| 0.58 | 0.24903474903474904 | 0.24281424281424285 | 0.06263406263406267 | -0.297726298 |
| 0.59 | 0.24511096462315976 | 0.22456602944407825 | 0.053944188090529574 | -0.329378159 |
| 0.6 | 0.240990990990991 | 0.22072072072072074 | 0.04054054054054057 | -0.362612613 |
| 0.61 | 0.23665973665973666 | 0.21667821667821668 | 0.027489027489027484 | -0.397551398 |
| 0.62 | 0.2321005215742058 | 0.21242294926505453 | 0.006401137980085347 | -0.434329066 |
| 0.63 | 0.22729486242999758 | 0.20793766739712688 | -0.007791575 | -0.473094716 |
| 0.64 | 0.2222222222222222 | 0.2032032032032032 | -1.39E-17 | -0.514014014 |
| 0.65 | 0.21685971685971683 | 0.19819819819819817 | -0.003861004 | -0.557271557 |
| 0.66 | 0.2111817700052994 | 0.19289878113407524 | -0.011393747 | -0.603073662 |
| 0.67 | 0.20515970515970514 | 0.18727818727818726 | -0.023478023 | -0.651651652 |
| 0.68 | 0.19876126126126123 | 0.1813063063063063 | -0.016328829 | -0.703265766 |
| 0.6900000000000001 | 0.19195001453065963 | 0.17494914269107814 | -0.027608253 | -0.758209823 |
| 0.7000000000000001 | 0.2282282282282282 | 0.16816816816816815 | -0.01951952 | -0.816816817 |
| 0.71 | 0.22305063684374027 | 0.16091954022988508 | -0.026095061 | -0.879465673 |
| 0.72 | 0.2175032175032175 | 0.185971685971686 | -0.021235521 | -0.946589447 |
| 0.73 | 0.21154487821154488 | 0.1800133466800134 | -0.019853187 | -1.018685352 |
| 0.74 | 0.20512820512820512 | 0.17359667359667363 | -0.021136521 | -1.096327096 |
| 0.75 | 0.19819819819819817 | 0.16666666666666669 | -0.022522523 | -1.18018018 |
| 0.76 | 0.19069069069069067 | 0.15915915915915918 | -0.024024024 | -1.271021021 |
| 0.77 | 0.1825303564433999 | 0.15099882491186842 | -0.025656091 | -1.369761065 |
| 0.78 | 0.1736281736281736 | 0.1420966420966421 | -0.027436527 | -1.477477477 |
| 0.79 | 0.16387816387816384 | 0.13234663234663235 | -0.029386529 | -1.595452595 |
| 0.8 | 0.15315315315315312 | 0.12162162162162163 | -0.031531532 | -1.725225225 |
| 0.81 | 0.1412991939307728 | 0.10976766239924132 | -0.033902323 | -1.868658132 |
| 0.8200000000000001 | 0.12812812812812804 | 0.12812812812812813 | -0.016016016 | -2.028028028 |
| 0.8300000000000001 | 0.14016958134605192 | 0.1266560678325384 | 0.0045045045045045045 | -2.206147324 |
| 0.84 | 0.13851351351351354 | 0.125 | 0 | -2.406531532 |
| 0.85 | 0.13663663663663664 | 0.12312312312312314 | 0 | -2.633633634 |
| 0.86 | 0.1344916344916345 | 0.120978120978121 | 0 | -2.893178893 |
| 0.87 | 0.13201663201663202 | 0.11850311850311851 | 0 | -3.192654193 |
| 0.88 | 0.12912912912912913 | 0.11561561561561562 | 0 | -3.542042042 |
| 0.89 | 0.12571662571662573 | 0.07166257166257167 | 0 | -3.954954955 |
| 0.9 | 0.12162162162162163 | 0.06756756756756757 | 0 | -4.45045045 |
| 0.91 | 0.06706706706706705 | 0.06256256256256255 | 0 | -5.056056056 |
| 0.92 | 0.06081081081081078 | 0.05630630630630628 | 0 | -5.813063063 |
| 0.93 | 0.05276705276705273 | 0.04826254826254823 | 0 | -6.786357786 |
| 0.9400000000000001 | 0.04204204204204197 | 0.0990990990990991 | 0 | -8.084084084 |
| 0.9500000000000001 | 0.027027027027026904 | 0.0990990990990991 | 0 | -9.900900901 |
| 0.96 | 0.1036036036036036 | 0.0990990990990991 | 0 | -12.62612613 |
| 0.97 | 0.1036036036036036 | 0 | 0 | -17.16816817 |
| 0.98 | 0 | 0 | 0 | -26.25225225 |
| 0.99 | 0 | 0 | 0 | -53.5045045 |

Net benefit values are reported for threshold probabilities from 0.01 to 0.99 for three models (Kaiser, Kaiser+, ADC); the treat-all reference is provided, while treat-none is zero (shown as dotted line). Across clinically relevant thresholds for BI-RADS 4 (≈0.10–0.50), Kaiser and Kaiser+ consistently yield higher net benefit than ADC and both reference strategies. The ADC model's net benefit falls below zero at higher thresholds (≈0.63 and above), indicating harm relative to treat-none. Net benefit was computed as TP/N − FP/N × pt/(1−pt) for each threshold pt.

**Supplement Table 5. Diagnostic errors by pathological subtype at fixed threshold (t=5)**

**A. Overall false-positive (benign) and false-negative (malignant) rates with 95% CI**

| **Category** | **Model** | **n/N** | **Rate (%)** | **95% CI (%)** |
| --- | --- | --- | --- | --- |
| Benign (FP) | Kaiser | 24/121 | 19.8 | 13.7–27.8 |
| Benign (FP) | Kaiser+ | 21/121 | 17.4 | 11.6–25.1 |
| Benign (FP) | ADC | 80/121 | 66.1 | 57.3–73.9 |
| Malignant (FN) | Kaiser | 9/101 | 8.9 | 4.8–16.1 |
| Malignant (FN) | Kaiser+ | 15/101 | 14.9 | 9.2–23.1 |
| Malignant (FN) | ADC | 11/101 | 10.9 | 6.2–18.5 |

**B. Paired comparisons (McNemar) for false positives (benign subset)**

| **Comparison** | **Model1 FP only** | **Model2 FP only** | **p value** |
| --- | --- | --- | --- |
| Kaiser vs ADC | 3 | 59 | 2.85e-12 |
| Kaiser+ vs ADC | 0 | 59 | 4.32e-14 |
| Kaiser vs Kaiser+ | 3 | 0 | 0.248 |

**C. Paired comparisons (McNemar) for false negatives (malignant subset)**

| **Comparison** | **Model1 FN only** | **Model2 FN only** | **p value** |
| --- | --- | --- | --- |
| Kaiser vs ADC | 7 | 9 | 0.803 |
| Kaiser+ vs ADC | 7 | 3 | 0.343 |
| Kaiser vs Kaiser+ | 0 | 6 | 0.041 |

(A) Overall error rates: FP among benign lesions (denominator n = 121) and FN among malignant lesions (denominator n = 101), with 95% confidence intervals.
(B–C) Paired comparisons (McNemar tests): within benign and malignant subsets, counts of Model 1 only vs Model 2 only errors and corresponding two-sided *p* values (multiple-comparison adjustment as specified in Methods). In each comparison row, Model 1 refers to the first-named model and Model 2 refers to the second-named model (e.g., for "Kaiser vs ADC", Model 1 = Kaiser, Model 2 = ADC).
Decision rules**:** Kaiser/Kaiser+ are positive (malignant) at score ≥ 5; ADC rule is positive at ADC ≤ 1.4 × 10⁻³ mm²/s; Kaiser+ = Kaiser − 4 points when ADC > 1.4 × 10⁻³ mm²/s, otherwise unchanged.
Definitions: FP = benign lesion classified as malignant; FN = malignant lesion classified as benign. All analyses are per lesion and paired across models.

**Supplementary Table 6 Confusion matrices and analysis of potentially avoidable biopsies (overall, mass, and NME).**

| **Lesion Type** | **Model** | **TP** | **FP** | **FN** | **TN** | **Sensitivity (%)** | **Specificity (%)** | **PPV (%)** | **NPV (%)** | **Biopsy Avoidance Rate (%)** | **Potentially Avoidable Biopsies** |
| --- | --- | --- | --- | --- | --- | --- | --- | --- | --- | --- | --- |
| **Overall (n=222)** |  |  |  |  |  |  |  |  |  |  |  |
|  | Kaiser (≥5) | 92 | 24 | 9 | 97 | 91.1 | 80.2 | 79.3 | 91.5 | 80.2 | 97/121 |
|  | Kaiser+ (≥5) | 86 | 21 | 15 | 100 | 85.1 | 82.6 | 80.4 | 87.0 | 82.6 | 100/121 |
|  | ADC (≤1.4) | 90 | 80 | 11 | 41 | 89.1 | 33.9 | 52.9 | 78.8 | 33.9 | 41/121 |
| **Mass Lesions (n=165)** |  |  |  |  |  |  |  |  |  |  |  |
|  | Kaiser (≥5) | 65 | 14 | 6 | 80 | 91.5 | 85.1 | 82.3 | 93.0 | 85.1 | 80/94 |
|  | Kaiser+ (≥5) | 62 | 13 | 9 | 81 | 87.3 | 86.2 | 82.7 | 90.0% | 86.2 | 81/94 |
|  | ADC (≤1.4) | 61 | 60 | 10 | 34 | 85.9 | 36.2 | 50.4 | 77.3 | 36.2 | 34/94 |
| **NME Lesions (n=57)** |  |  |  |  |  |  |  |  |  |  |  |
|  | Kaiser (≥5) | 27 | 10 | 3 | 17 | 90.0 | 63.0 | 73.0 | 85.0 | 63.0 | 17/27 |
|  | Kaiser+ (≥5) | 26 | 8 | 4 | 19 | 86.7 | 70.4 | 76.5 | 82.6 | 70.4 | 19/27 |
|  | ADC (≤1.4) | 29 | 20 | 1 | 7 | 96.7 | 25.9 | 59.2 | 87.5 | 25.9 | 7/27 |

TP = True Positive; FP = False Positive; FN = False Negative; TN = True Negative. Biopsy Avoidance Rate = TN/(TN+FP), representing the proportion of benign lesions correctly classified as benign (equivalent to specificity). Diagnostic thresholds: Kaiser/Kaiser+ ≥5, ADC <1.4 × 10⁻³ mm²/s. Sample composition: Overall n=222 (121 benign, 101 malignant), Mass n=165 (94 benign, 71 malignant), NME n=57 (27 benign, 30 malignant).

**Supplement Table 7 False-Negative and False-Positive Errors by Pathological Subtype for Three Diagnostic Models at Fixed Threshold (t=5)**

| **Pathology** | **N(Total)** | **N (Malignant)** | **N (Benign)** | **Kaiser FN** | **Kaiser+ FN** | **ADC FN** | **Kaiser FP** | **Kaiser+ FP** | **ADC FP** |
| --- | --- | --- | --- | --- | --- | --- | --- | --- | --- |
| Fibroadenoma | 54 | 0 | 54 | N/A | N/A | N/A | 8/54 (14.8%) | 8/54 (14.8%) | 32/54 (59.3%) |
| Breast Hyperplasia | 39 | 0 | 39 | N/A | N/A | N/A | 9/39 (23.1%) | 7/39 (17.9%) | 27/39 (69.2%) |
| Intraductal Papilloma | 23 | 3 | 20 | 1/3 (33.3%) | 1/3 (33.3%) | 0/3 (0.0%) | 5/20 (25.0%) | 4/20 (20.0%) | 14/20 (70.0%) |
| Mucinous carcinoma of the breast | 6 | 6 | 0 | 1/6 (16.7%) | 4/6 (66.7%) | 5/6 (83.3%) | N/A | N/A | N/A |
| DCIS | 21 | 21 | 0 | 3/21 (14.3%) | 4/21 (19.0%) | 2/21 (9.5%) | N/A | N/A | N/A |
| IDC | 58 | 58 | 0 | 1/58 (1.7%) | 2/58 (3.4%) | 2/58 (3.4%) | N/A | N/A | N/A |
| Mastitis | 4 | 0 | 4 | N/A | N/A | N/A | 1/4 (25.0%) | 1/4 (25.0%) | 3/4 (75.0%) |
| Benign phyllodes tumor of the breast | 3 | 0 | 3 | N/A | N/A | N/A | 1/3 (33.3%) | 1/3 (33.3%) | 3/3 (100.0%) |
| ILC | 2 | 2 | 0 | 1/2 (50.0%) | 1/2 (50.0%) | 0/2 (0.0%) | N/A | N/A | N/A |
| Borderline phyllodes tumor of the breast | 1 | 1 | 0 | 1/1 (100.0%) | 1/1 (100.0%) | 0/1 (0.0%) | N/A | N/A | N/A |
| Malignant phyllodes tumor of the breast | 1 | 1 | 0 | 0/1 (0.0%) | 1/1 (100.0%) | 1/1 (100.0%) | N/A | N/A | N/A |
| Metaplastic carcinoma of the breast with mesenchymal differentiation | 1 | 1 | 0 | 1/1 (100.0%) | 1/1 (100.0%) | 0/1 (0.0%) | N/A | N/A | N/A |
| Leiomyoma of the breast | 1 | 0 | 1 | N/A | N/A | N/A | 0/1 (0.0%) | 0/1 (0.0%) | 1/1 (100.0%) |
| Breast DLBCL | 1 | 1 | 0 | 0/1 (0.0%) | 0/1 (0.0%) | 1/1 (100.0%) | N/A | N/A | N/A |
| Inflammatory myofibroblastic tumor | 1 | 1 | 0 | 0/1 (0.0%) | 0/1 (0.0%) | 0/1 (0.0%) | N/A | N/A | N/A |
| Invasive apocrine carcinoma | 2 | 2 | 0 | 0/2 (0.0%) | 0/2 (0.0%) | 0/2 (0.0%) | N/A | N/A | N/A |
| Malignant phyllodes tumor | 1 | 1 | 0 | 0/1 (0.0%) | 0/1 (0.0%) | 0/1 (0.0%) | N/A | N/A | N/A |
| Glycogen-rich clear cell carcinoma | 1 | 1 | 0 | 0/1 (0.0%) | 0/1 (0.0%) | 0/1 (0.0%) | N/A | N/A | N/A |
| Mixed tubular carcinoma | 1 | 1 | 0 | 0/1 (0.0%) | 0/1 (0.0%) | 0/1 (0.0%) | N/A | N/A | N/A |
| Solid papillary carcinoma of the breast | 1 | 1 | 0 | 0/1 (0.0%) | 0/1 (0.0%) | 0/1 (0.0%) | N/A | N/A | N/A |

Distribution of FN and FP errors across pathological subtypes for three diagnostic models at a fixed classification threshold (t=5). All metrics were computed using threshold-based binary classification: Kaiser and Kaiser+ scores ≥5% classified as BI-RADS ≥4 (malignant); ADC values ≤1.4 mm²/s classified as malignant.
